# The Study of Plasticized Solid Polymer Blend Electrolytes Based on Natural Polymers and Their Application for Energy Storage EDLC Devices

**DOI:** 10.3390/polym12112531

**Published:** 2020-10-29

**Authors:** Elham M.A. Dannoun, Shujahadeen B. Aziz, Mohamad A. Brza, Muaffaq M. Nofal, Ahmad S.F.M. Asnawi, Yuhanees M. Yusof, Shakhawan Al-Zangana, Muhamad H. Hamsan, Mohd F. Z. Kadir, Haw J. Woo

**Affiliations:** 1 Associate Director of General Science Department, Woman Campus, Prince Sultan University, P. O. Box 66833, Riyadh 11586, Saudi Arabia; elhamdannoun1977@gmail.com; 2 Hameed Majid Advanced Polymeric Materials Research Lab., Physics, College of Science, University of Sulaimani, Qlyasan Street, Sulaimani 46001, Kurdistan Regional Government, Iraq; 3 Department of Civil engineering, College of Engineering, Komar University of Science and Technology, Sulaimani 46001, Kurdistan Regional Government, Iraq; 4 Manufacturing and Materials Engineering Department, Faculty of Engineering, International Islamic University of Malaysia, Kuala Lumpur 50603, Gombak, Malaysia; mohamad.brza@gmail.com; 5 Department of Mathematics and General Sciences, Prince Sultan University, P. O. Box 66833, Riyadh 11586, Saudi Arabia; muaffaqnofal@gmail.com; 6 Chemical Engineering Section, Universiti Kuala Lumpur, Malaysian Institute of Chemical & Bioengineering Technology (UniKL MICET), Alor Gajah 78000, Malacca, Malaysia; asyafiq.asnawi@s.unikl.edu.my (A.S.F.M.A.); yuhanees@unikl.edu.my (Y.M.Y.); 7 Department of Physics, College of Education, University of Garmian, Kalar 46021, Iraq; shakhawan.al-zangana@garmian.edu.krd; 8 Institute for Advanced Studies, University of Malaya, Kuala Lumpur 50603, Malaysia; hafizhamsan93@gmail.com; 9 Centre for Foundation Studies in Science, University of Malaya, Kuala Lumpur 50603, Malaysia; mfzkadir@um.edu.my; 10 Centre for Ionics, Department of Physics, Faculty of Science, University of Malaya, Kuala Lumpur 50603, Malaysia; woohj@um.edu.my

**Keywords:** polymer blend, magnesium salt, electrochemical impedance spectroscopy, cyclic voltammetry study, electrochemical double-layer capacitor

## Abstract

In this work, plasticized magnesium ion-conducting polymer blend electrolytes based on chitosan:methylcellulose (CS:MC) were prepared using a solution cast technique. Magnesium acetate [Mg(CH_3_COO)_2_] was used as a source of the ions. Nickel metal-complex [Ni(II)-complex)] was employed to expand the amorphous phase. For the ions dissociation enhancement, glycerol plasticizer was also engaged. Incorporating 42 wt% of the glycerol into the electrolyte system has been shown to improve the conductivity to 1.02 × 10^−4^ S cm^−1^. X-ray diffraction (XRD) analysis showed that the electrolyte with the highest conductivity has a minimum crystallinity degree. The ionic transference number was estimated to be more than the electronic transference number. It is concluded that in CS:MC:Mg(CH_3_COO)_2_:Ni(II)-complex:glycerol, ions are the primary charge carriers. Results from linear sweep voltammetry (LSV) showed electrochemical stability to be 2.48 V. An electric double-layer capacitor (EDLC) based on activated carbon electrode and a prepared solid polymer electrolyte was constructed. The EDLC cell was then analyzed by cyclic voltammetry (CV) and galvanostatic charge–discharge methods. The CV test disclosed rectangular shapes with slight distortion, and there was no appearance of any redox currents on both anodic and cathodic parts, signifying a typical behavior of EDLC. The EDLC cell indicated a good cyclability of about (95%) for throughout of 200 cycles with a specific capacitance of 47.4 F/g.

## 1. Introduction

The new characteristics of nano-sized metal particles (Fe, Ce, Cu, Zn and Ni) are nowadays studied because of their large surface areas and electronic structure. Metal complexes have emerged as an area of great current interest motivated by possible energy technology applications, electronics, optics, chemical catalysis and magnetics [1,2,3]. Their properties may be adjusted via control of the metal particle size, shape and organization. Furthermore, metal nano-particles also depend on the microenvironment’s chemical nature surrounding the particle [4]. As stated in the literature [5], metal complexes reduce the polymer electrolyte energy bandgap. This benefit is useful in energy device applications. Based on our previous work [6], the incorporation of Zn metal complex (Zn(II)-complex) has enhanced the amorphousness of chitosan-NH_4_F-glycerol electrolyte. Furthermore, the authors used the electrolyte in the electrochemical double-layer capacitor (EDLC) device application, and they indicated that the Ni(II)-complex improved the EDLC device performance. Brza et al. [5] documented that a Cu(II)-complex in polyvinyl alcohol (PVA) greatly enhanced the amorphous phase. This condition is advantageous to electrolyte application because ions are transported easily in the amorphous region, thus improving the ionic conductivity [3].

Biopolymers are natural polymers where various kinds of source can be used. Biopolymers usually are inexpensive, naturally abundant, have high compatibility with solvents and are very stable in forming a film [7,8]. A solid polymer electrolyte is considered as a suitable candidate for energy device applications, due to the good electrochemical and thermal stability, in addition to high ionic conductivity at ambient temperature [9,10,11,12]. Moreover, the good flexibility of this type of electrolyte enhances the arrangement in various geometries to create optimal electrode/electrolyte contact, which is crucial for energy devices [13,14]. Literature revealed that energy devices based on a polymer electrolyte have been widely addressed and researchers have put great effort into this with particular attention given to batteries [9,10,11,12,13,14,15,16] and supercapacitors (SCs) [17,18,19,20,21,22,23,24].

Many works on polymer electrolyte (PE) have reported that ionic conductivity ranged from 10^−5^ to 10^−3^ S/cm, where biopolymers were used as host polymers, such as cellulose, starch, dextran, chitin, gelatin, chitosan (CS) and carrageenan [25,26,27]. Chitosan is widely studied in energy device technologies and environmental and health approaches [28]. Ions from salt can be transported through the oxygen-containing functional groups present in the CS structure, such as hydroxyl (OH), glycosidic linkage, anhydroglucose ring oxygen and amine [29]. The inclusion of organic materials like methyl chloride or dimethyl sulfate to alkali-based cellulose produces a tailored biopolymer called methylcellulose (MC) with a 1,4 glycosidic bond [30].

For many decades, batteries have been the primary energy sources used in various electrical appliances and vehicles. The preparation of batteries is much more expensive and complicated than the alternative EDLC. Electrodes used in an EDLC are usually carbon-based, where ions from salt undergo a non-Faradaic process for storing energy [31]. Various types of altered carbons, such as graphite [32], carbon aerogel [33], carbon nanotubes [34] and activated carbon [35], have been used as the active material in EDLCs. Activated carbon has been extensively studied in the electrodes field due to its extra surface area (~2500 m^2^/g), good chemical stability and excellent electronic conductivity [36]. The EDLC is preferable to other SCs due to its high power density, high durability and better thermal stability, also being cheap with an uncomplicated methodology to make EDLC devices [37,38,39].

In PE, it is noticeable that few works reported on magnesium salt-based PEs compared to Li^+^ or Na^+^ or H^+^ [40]. Low equivalence weight, low price, high safety and high reduction potential compared to lithium are why magnesium salt-based PEs can be an excellent substitute for other types of electrolyte [41]. The work of Polu et al. [42] showed that the inclusion of magnesium salt enhanced the ionic conductivity and flexibility of PVA as the glass transition temperature (*T*_g_) of the polymer decreased. Hassan et al. [43] concluded that ionic diffusivity, number density and mobility had been improved by incorporating 35 wt% magnesium sulfate (MgSO_4_). In this work, CS is blended with MC to produce a more stable polymer host. Magnesium acetate Mg(CH_3_COO)_2_ and glycerol are used as ionic sources and plasticizers, respectively. The effect of the addition of a Ni metal complex (Ni(II)-complex) on PE electrical and structural properties has also been investigated. The highest conducting electrolyte is used in the fabrication of the EDLC device.

## 2. Materials and Methods

### 2.1. Materials and Sample Preparation

All chemicals were obtained from Sigma-Aldrich (St. Louis, MO, USA) and used with no extra purification. Two primary raw materials were used to fabricate glycerolized CS:MC:Mg(CH_3_COO)_2_:Ni(II)-complex blend electrolyte systems, CS and MC with the average molecular weights of 310,000–375,000 g/moL and 10,000–220,000 g/mol, respectively. Other raw materials were magnesium acetate (Mg(CH_3_COO)_2_), acetic acid (CH_3_COOH), Ni(II)-complex and glycerol (C_3_H_8_O_3_), involved in the solution-casting technique. The blend’s fabrication contained a dissolution of 0.5 g of CS in 30 mL of the 1% acetic acid solution, which was then stirred for several hours at ambient temperature. The MC solution was prepared by dissolving and stirring 1 g of MC in 80 mL distilled water for several hours by a magnetic stirrer until a homogeneous solution was achieved at room temperature. The two different solutions (i.e., CS and MC) were combined and continuously stirred with a magnetic stirrer to fabricate CS:MC polymer blends. Next, 40 wt% of Mg(CH_3_COO)_2_ salt was incorporated and stirred into the CS:MC blended solutions until a homogenous solution was achieved. Additionally, 10 mL of Ni(II)-complex of a fixed amount was added to the electrolyte systems. Finally, glycerol separately with different amounts was added to the electrolyte systems. Then, the CS:MC:Mg(CH_3_COO)_2_:Ni(II)-complex systems were coded as CSMCMNG1, CSMCMNG2 and CSMCMNG3 with the addition of 14, 28 and 42 wt% of glycerol, respectively. Each fabricated solution was then poured into clean and dry labeled Petri dishes and left to evaporate at ambient temperature. Table 1 displays the composition of the electrolyte films.

### 2.2. X-ray Diffraction

For X-ray diffraction (XRD) experiments, a Siemens D5000 X-ray diffractometer (Bruker AXS GmbH, Berlin, Germany) was applied under a certain electric operating condition (40 kV and 40 mA). Monochromatic CuKα radiation (1.5406 Ǻ) at glancing angles between 10° and 80° with a step size of 0.1° was scanned over the films. To obtain the crystallinity degree, it is crucial to deconvolute each film XRD pattern to determine the areas of the amorphous and crystalline peaks [44]. The degree of crystallinity (*X_c_*) was calculated with Equation (1) [45]:

(1) $$Xc=\frac{A_{C}}{A_{T}}\times 100\%$$

where *A_C_* and *A_T_* refer to the crystalline peaks area and the total area of crystalline and amorphous peaks, respectively.

### 2.3. Electrical Impedance Spectroscopy (EIS)

Electrical impedance spectroscopy (EIS) is an appropriate technique for learning a material’s electrical property, and has been used in electrochemical energy storage devices [46]. It gives vital evidence about the electrical properties at the interfacial space among electronically conducting electrodes and electrolytes. Before impedance measurements, the electrolyte films were cut into small discs of 2 cm in diameter and then positioned between two stainless steel electrodes via spring pressure. The impedance measurements were performed via HIOKI 3531 Z Hi-tester (Hioki, Nagano, Japan) with a computer in the frequency range between 50 Hz and 5000 kHz at room temperature. Software extracted both real (*Z′*) and imaginary (*Z″*) parts of the impedance spectra of the EIS plots. From the plot’s intersection with the real axis, the bulk resistance (*R_b_*) was obtained. From the *R_b_*, the conductivity of the films was measured using Equation (2) [46]:

(2) $$\sigma_{dc}=\left(\frac{1}{R_{b}}\right)\times \left(\frac{t}{A}\right)$$

where *t* is the thickness of the film and *A* is the area of the electrode.

### 2.4. Electrochemical Characterization

#### 2.4.1. Linear Sweep Voltammetry (LSV) and Transference Number Measurements (TNMs)

Linear sweep voltammetry (LSV) was conducted to test the cells’ electrochemical stability, using Digi-IVY DY2300 potentiostat (Neware, Shenzhen, China) at a scan rate of 20 mV/s. Transference number measurements (TNMs) were performed at room temperature using a direct current (DC) polarization method by tracking the polarization current as a function of time. The cell was polarized at 0.2 V using a V&A Instrument DP3003 digital DC power supply (V & A Instrument, Shanghai, China). For both TNM and LSV, the cell consisted of the highest conducting polymer blend electrolyte (CSMCMNG3) sandwiched by two stainless steel electrodes (SSE). Ionic transference number (*t_i_*) can be determined if the initial (*I_i_*) and steady-state (*I_ss_*) currents are known using the following Equation (3):

(3) $$t_{i}=\frac{I_{i}-I_{ss}}{I_{i}}$$

The following Equation (4) can obtain electron transference number (*t_e_*):

(4) $$t_{e}=1-t_{i}$$

#### 2.4.2. Fabrication and Characterization of Electric Double-Layer Capacitor (EDLC)

The carbon electrodes preparation was the initial step for the EDLC preparation. Activated carbon of 3.25 g was dry-mixed with 0.25 g carbon black powder, using a planetary ball miller (XQM-0.4) at a rotational speed of 500 r/min for 15 min. Moreover, 0.50 g polyvinylidene fluoride (PVdF) was dissolved in 15 mL N-methyl pyrrolidone (NMP) at room temperature. The activated carbon-carbon black powders were then dissolved for 90 min in the PVdF-NMP solution. The obtained homogeneous solution was then coated on aluminum foil and dried in an oven at 60 °C for 120 min. Lastly, the dried electrodes (thickness = 0.01 cm) were stored in a desiccator filled with silica gel to remove extra moisture. The highest conducting electrolyte cut into a circle shape with an area of 2.01 cm^2^ was sandwiched between two carbon electrodes prepared in the initial step and packed in CR2032 coin cells. The galvanostatic charge–discharge characteristics of the EDLC were conducted using a NEWARE battery cycler (current density = 0.75 mA/cm^2^). Several parameters of the EDLC can be obtained using the following Equations (5)–(9):

(5) $$C_{cd}=\frac{i}{gm}$$

(6) $$ESR=\frac{V_{d}}{i_{}}$$

(7) $$E=\frac{C_{s}V^{2}}{2}$$

(8) $$P_{}=\frac{V^{2}}{4m(ESR)}$$

(9) $$Efficiency=\frac{t_{dis}}{t_{cha}}$$

where *i* is the applied current, which was 1.5 mA, *g* is the slope of the discharge part, *m* is the mass of active material, *V_d_* is the voltage drop before the discharge process initiates and *V* is the voltage applied (0.9 V). *t_d_* and *t_c_* are the time taken for one complete discharging and charging, respectively. A Digi-IVY DY2300 Potentiostat was used to perform cyclic voltammetry (CV) analysis of the EDLC at different scan rates (from 10 to 100 mV/s). The potential range was from 0 to 0.9 V. Specific capacitance (*C_cv_*) can be obtained from CV using the following Equation (10):

(10) $$C_{cv}=\int_{V_{i}}^{V_{f}}\frac{I\left(V\right)dV}{2mh\left(V_{f}-V_{i}\right)}$$

where *I(V)dV* stands for the CV’s area determined using the Origin 9.0 software through the integration function. *m* and *h* are the mass of active material and scan rate, respectively. *V_i_* and *V_f_* in this work are 0 V and 0.9 V, respectively.

## 3. Results and Discussion

### 3.1. X-ray Diffraction (XRD) Study

**Figure 1:**
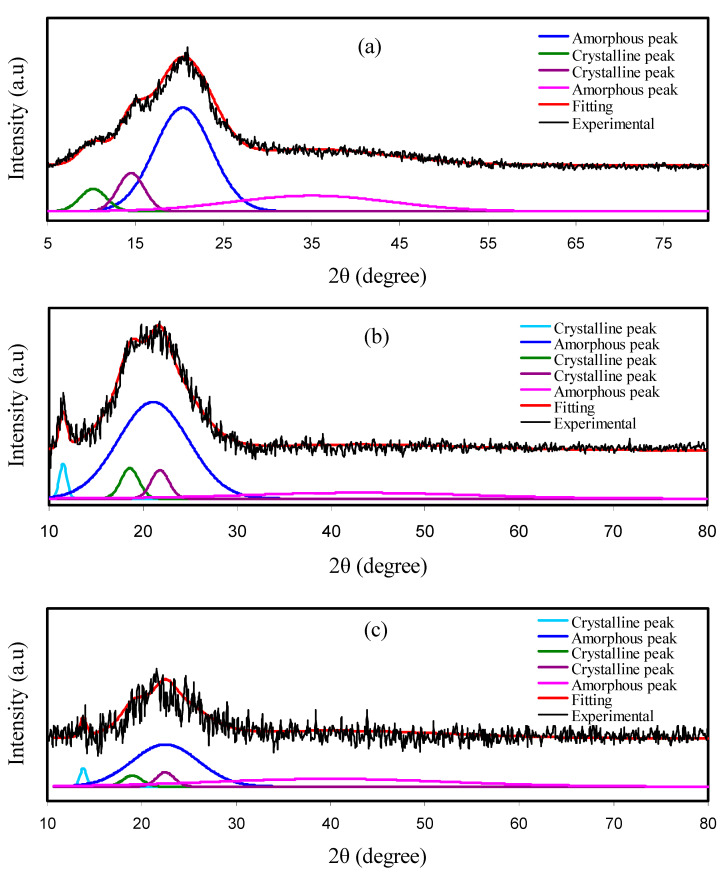

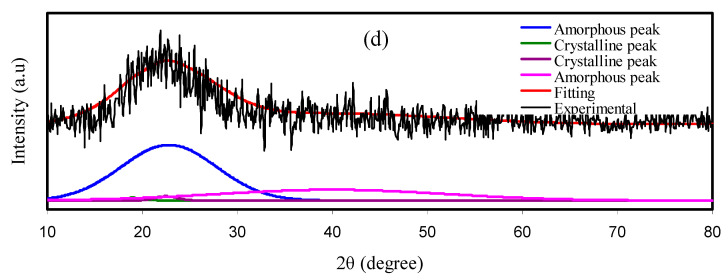
XRD spectra for (**a**) pure CS, (**b**) CS:MC, (**c**) CSMCMNG1 and (**d**) CSMCMNG3 electrolyte films.

Figure 1 shows the deconvoluted XRD spectra for the electrolyte films. The XRD spectrum of pure CS film is pointed out in Figure 1a. It is accepted that the crystalline peaks for pure CS are recorded at 2θ = 15.1, 17.7 and 20.9° as a consequence of inter- and intra-hydrogen bonding between the functional groups of individual monomers and the chains [44,47]. The MC semi-crystalline nature is an inherent structural feature that enables X-ray examinations to be performed through the MC’s influence on the biopolymer matrix [48]. It has been reported that there exists only one hollow at 2θ = 19°–21° for MC material, which originates from the intermolecular hydrogen bonding together with a short-distance order in the MC polymer chains [49,50,51]. The interesting point is that a broad peak at a diffraction angle 2θ of 8.03° is recognized to relate to the presence of tri-methyl glucose repeating unit within the MC [48].

It should be noted that two distinct concave peaks can be seen from the XRD pattern of the CS:MC system (see Figure 1b). The broad hollows can verify that the CS:MC blend indicates an entirely amorphous structure [52,53]. Figure 1c, d shows the XRD pattern obtained for the desired blend electrolyte samples. Interestingly, with the addition of 14 wt% of glycerol plasticizer into CS:MC:Mg(CH_3_COO)_2_:Ni(II)-complex matrix, the crystalline peaks and hollow intensity were lowered as can be seen in Figure 1c. This can be considered as evidence of decreasing the crystalline region in the CS matrix [44]. It is obvious from Figure 1d that adding 42 wt% of plasticizer to CS:MC mixture can result in more broadening of the hollow and noticeably decreasing the crystalline diffraction peaks. Peak broadening and lowering in intensity reveal that the amorphous region within the blended polymer is dominant. Another advantage of XRD analysis is that it can anticipate the electrolyte’s conductivity trend [54].

The Xc for each system was calculated using Equation (1). It is remarkable that the Xc is decreased upon the insertion of MC content (see Table 2). The deconvoluted XRD spectra of plasticized CS:MC electrolyte is shown in Figure 1c,d. It is interesting to note that the intensity of the XRD peaks is considerably reduced. Based on former research, salt can terminate the polymer chain’s hydrogen bonding because of electrostatic reactions created among the salt cations and polymer functional groups [55]. The amorphous structure growth might be related to the crystalline phase interruption in the polymer [56]. Compared to the pure blend systems, the plasticized system’s degree of crystallinity is very significantly diminished (see Table 2). Plasticizers are a famous approach to raising the number of free ions and decreasing the crystallinity [57].

### 3.2. Impedance Analysis

**Figure 2:**
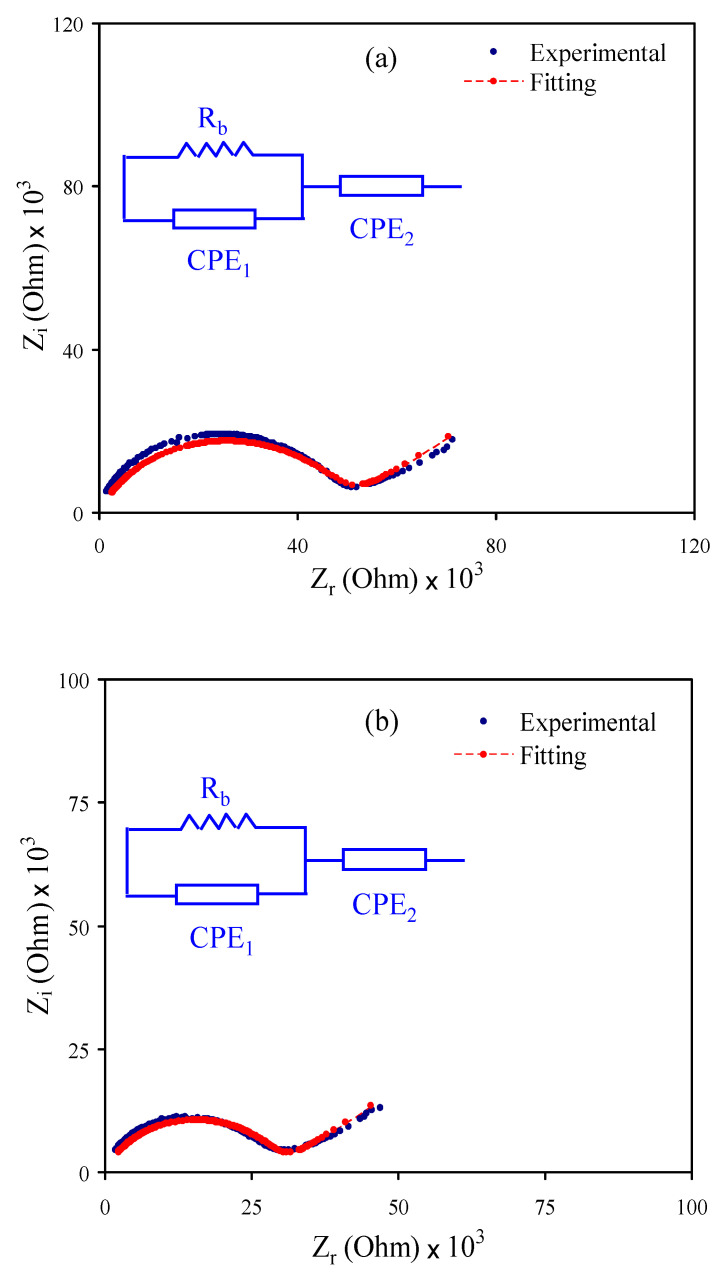

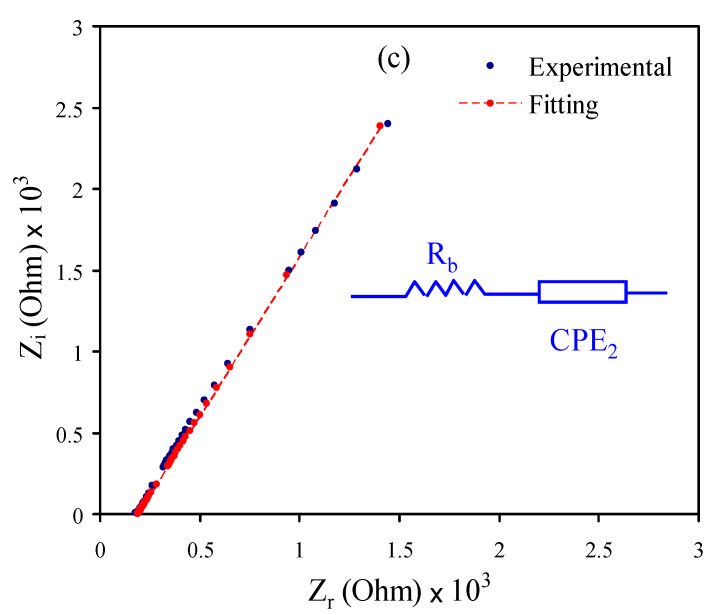
Electrochemical impedance spectroscopy (EIS) plots for (**a**) CSMCMNG1, (**b**) CSMCMNG2 and (**c**) CSMCMNG3 electrolyte films.

Using electrochemical impedance spectroscopy (EIS), the electrochemical and ion transference behaviors of ionic materials, such as electrodes and PEs, can be examined, effectively [53,58,59,60,61]. Here, EIS was used to analyze the electrolyte films shown in Figure 2a–c. For CSMCMNG1 and CSMCMNG2 systems shown in Figure 2a,b, a semicircle at the high-frequency region has resulted from the bulk effect of the electrolytes, and a tail at low frequencies can be noted. Due to the creation of EDLC, the tail at the low frequencies occurs through the free charges’ accumulation at the electrode and electrolyte interface [62]. The CSMCMNG3 system only displayed the spike or tail (see Figure 2c).

As a straightforward method for EIS examination, the electrical equivalent circuit (EEC) method is used, which provides the entire image of the electrolyte system [63]. In terms of the EEC, the Nyquist plot for the systems was deduced. It comprises bulk resistance (*R_b_*) for the electrolyte system carriers and two constant phase elements (CPE), as shown in the Figure 2 inset. The high-frequency region displays the parallel connection of *R_b_* and constant phase element, while the low-frequency region indicates only the constant phase element, meaning that the EDLC formed between electrode and electrolyte. In EEC, the constant phase elements are used more generally than the ideal capacitor in the real system. *Z_CPE_* impedance can be written as [53,64,65]:

(11) $$Z_{CPE}=\frac{1}{C\omega^{p}}\left[\cos\left(\frac{\pi p}{2}\right)-i\sin\left(\frac{\pi p}{2}\right)\right]$$

where *C* stands for the constant phase element’s capacitance, *ω* refers to the angular frequency and *p* is linked from the vertical axis to the EIS plot’s departure.

Here, the real (*Z_r_*) and imaginary (*Z_i_*) parts of complex impedance (Z*) correlated with the EEC (inset of Figure 2a,b) are indicated as [66]:

(12) $$Z_{r}=\frac{R_{b}C_{1}\omega^{p1}\cos\left(\frac{\pi p_{1}}{2}\right)+R_{b}}{2R_{b}C_{1}\omega^{p1}\cos\left(\frac{\pi p_{1}}{2}\right)+R_{b}{}^{2}C_{1}{}^{2}\omega^{2p1}+1}+\frac{\cos\left(\frac{\pi p_{2}}{2}\right)}{C_{2}\omega^{p2}}$$

(13) $$Z_{i}=\frac{R_{b}C_{1}\omega^{p1}\sin\left(\frac{\pi p_{1}}{2}\right)}{2R_{b}C_{1}\omega^{{}^{p1}}\cos\left(\frac{\pi p_{1}}{2}\right)+R_{b}{}^{2}C_{1}{}^{2}\omega^{2P1}+1}+\frac{\sin\left(\frac{\pi p_{2}}{2}\right)}{C_{2}\omega^{p2}}$$

where *C_1_* refers to the bulk constant phase element capacitance and *C_2_* refers to the constant phase element capacitance at the electrode and electrolyte interface.

Here, the *Z_r_* and *Z_i_* parts of Z^*^ associated with the EEC (inset of Figure 2c) are expressed as [66,67]:

(14) $$Z_{r}=R+\frac{\cos\left(\frac{\pi p_{2}}{2}\right)}{C_{2}\omega^{p2}}$$

(15) $$Z_{i}=\frac{\sin\left(\frac{\pi p_{2}}{2}\right)}{C_{2}\omega^{p2}}$$

Table 3 shows the fitting parameters of the EEC. *R_b_* is obtained by the interception between the real axis and the spike. It is evident that, upon the addition of glycerol, the semicircle at the high-frequency region was absent (see Figure 2c) due to the complete transport of ions toward the electrodes. To calculate the ionic conductivity of the electrolytes, Equation (2) was used. In the present work, the obtained maximum ionic conductivity is 1.02 × 10^−4^ S cm^−1^ when 42 wt% glycerol was added at room temperature, as listed in Table 4. *R_b_* decreased as the glycerol amount increased to 42 wt% due to increased charge carriers; thus, ion conductivity increases.

### 3.3. Electrochemical Studies

#### 3.3.1. Transference Number Measurement (TNM) Study

**Figure 3:**
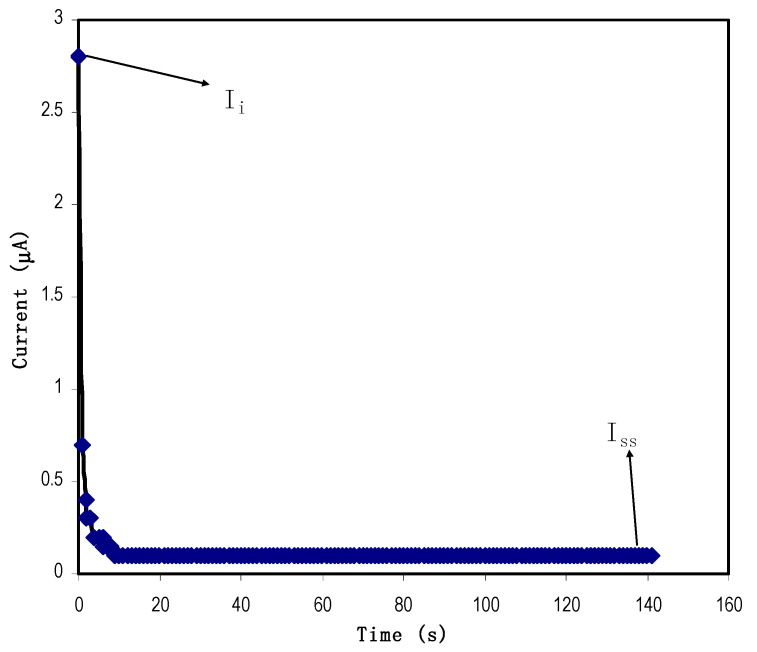
Polarization of the highest conducting electrolyte at 0.8 V working voltage.

Both ions and electrons carry electric charges in a PE system. For EDLC applications, it is essential to use a PE with high *t_i_* rather than high *t_e_.* The energy storage process of the EDLC is through adsorption/desorption of ions at the surface of carbon electrodes. TNM analysis has been conducted where the PE is subjected to a working voltage of 0.8 V. Figure 3 shows the polarization plot for the highest conducting electrolyte at room temperature. As the voltage is applied, both ions and electrons move towards the electrodes, producing a high *I_i_* of 2.8 μA. As time goes by, ions are blocked at the surface of the stainless steel (SS) electrodes. This situation reduces the current flow. Stable current (0.1 μA) can be observed beyond 10 s; it means that the PE is now completely polarized and at this point only electrons can flow through the SS electrodes. This phenomenon is the typical behavior of an ionic conductor accompanying electron transfer [68]. From Equations (3) and (4), *t_i_* and *t_e_* are identified as 0.964 and 0.036, respectively. *t_i_* is found to be more than the reported value of 0.95 for Mg(CH_3_COO)_2_, Mg(NO_3_)_2_ and MgCl_2_ [42,69,70]. Thus, *t_i_* value in this work approves the PE to be used in the EDLC fabrication.

#### 3.3.2. Linear Sweep Voltammetry (LSV) Study

**Figure 4:**
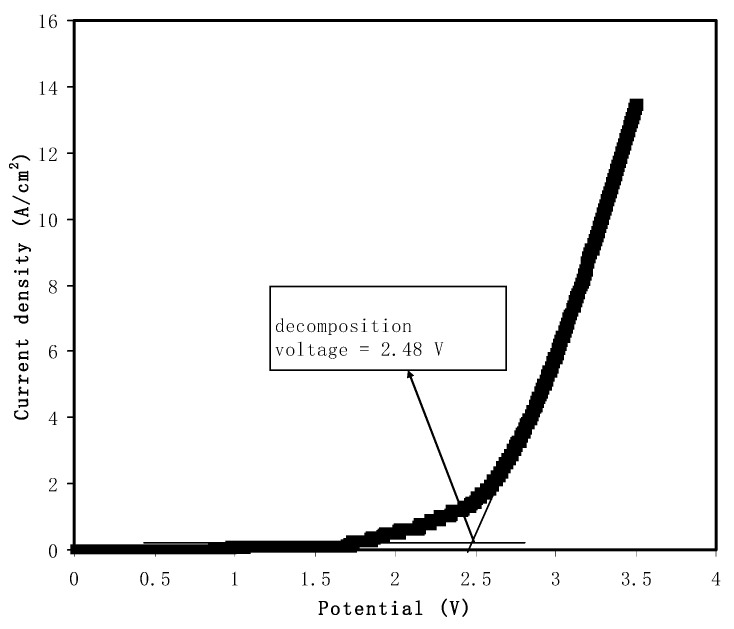
Linear sweep voltammetry (LSV) plot of the highest conducting electrolyte at 20 mV/s.

Linear sweep voltammetry (LSV) analysis was performed to study the potential stability of the PE by using a 20 mV/s scan rate. The potential stability of a PE is vital because, in energy storage devices, the rapid charge–discharge process can degrade the PE if the potential range is higher than the decomposition of the PE. LSV is a standard method used in the study of fuel cells, SCs, solar cells and batteries [71]. Figure 4 illustrates the current pattern as the potential is swept linear up to 3.5 V. A sharp increase of current from 2 to 14 mA/cm^2^ can be observed as the potential varies from 2.4 to 3.5 V. The drastic increase in current values indicates the degradation process of the polymer [72]. As reported by Jo et al. [73], a magnesium perchlorate (Mg(ClO_4_)_2_) system is electrochemically stable up to ~2 V. A film of PMMA-Mg(CF_3_SO_3_)_2_ is reported that can withstand potential up to 2.5 V [74]. Hence, it can be concluded that the highest conducting electrolyte in this work can be used as an electrodes separator in the fabrication of EDL.

#### 3.3.3. Cyclic Voltammetry (CV) Studies of Fabricated EDLC

**Figure 5:**
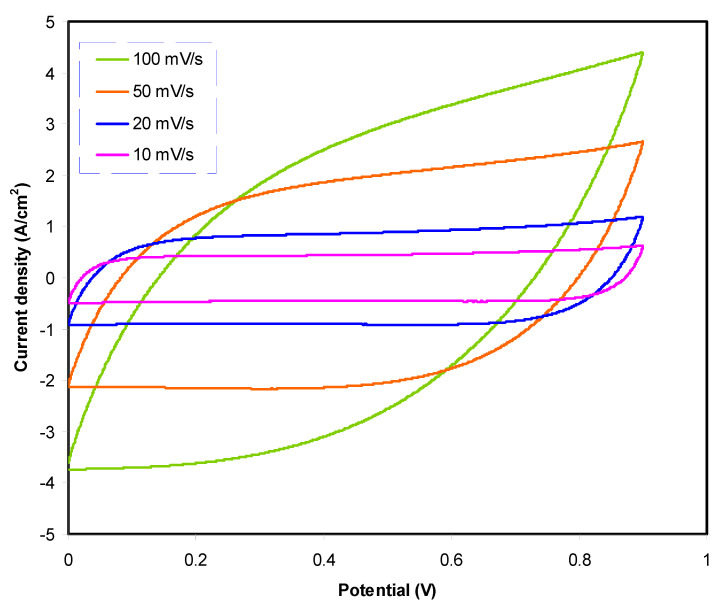
Cyclic voltammetry (CV) plot of the highest conducting electrolyte at various scan rates.

The constructed EDLC with the arrangement of activated carbon|highest conducting sample|activated carbon is analyzed using cyclic voltammetry (CV) analysis. Figure 5 shows CV plots with the absence of redox peaks. Peaks are usually shown in the CV plot of batteries or any energy devices requiring a chemical reaction with the electrode. Unlike batteries, ions undergo the adsorption/desorption process called non-Faradic [75]. CV shape for higher scan rate looks like a leaf, while at a lower scan rate the shape of the CV plot is more like a rectangular shape. One of the characteristics of a capacitor is that the CV response is scan rate dependent. At a large scan rate, the migration of ions occurs at a rapid rate.

Furthermore, due to carbon porosity and internal resistance, current-voltage dependence is produced [76]. The obtained value of *C_cv_* is tabulated in Table 5. At 100 mV/s, *C_cv_* is 16.8 F/g and 25.8 F/g as the scan rate reduces to 50 mV/s. *C_cv_* is observed to be highest at a low scan rate. These results show that, at a low scan rate, an almost perfect plateau region can be observed shown in Figure 5***,*** which indicates that the charge carriers move at an almost slow and stable rate, thus developing ion accumulation at the electrode–electrolyte boundary with low ohmic resistance [77]. The reduction of *C_cv_* with increasing scan rate is related to the presence of internal resistance. The time scale of the current to reach a horizontal constant value on the reversal of the potential scan is increased at a high scan rate. The longer delay at the switching potential induces the electrical double-layers’ slow reorganization due to the high resistance of ionic mobility in the micropores of activated carbon in the EDLC [78,79].

#### 3.3.4. Charge–Discharge Study

**Figure 6:**
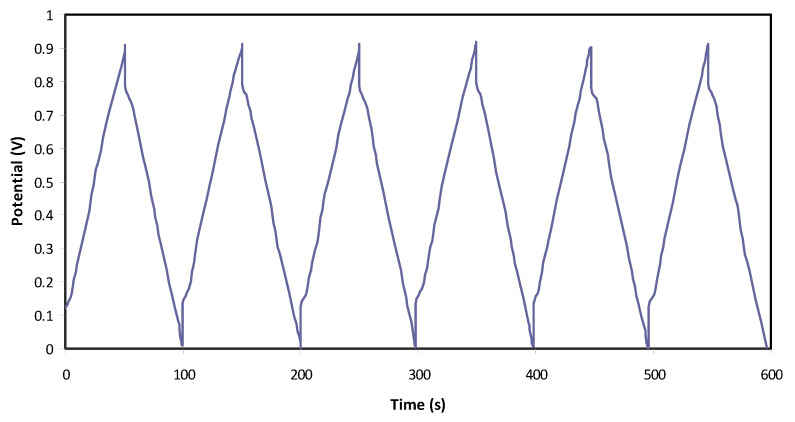
Charge–discharge plot of the electric double-layer capacitor (EDLC) at selected cycles.

**Figure 7:**
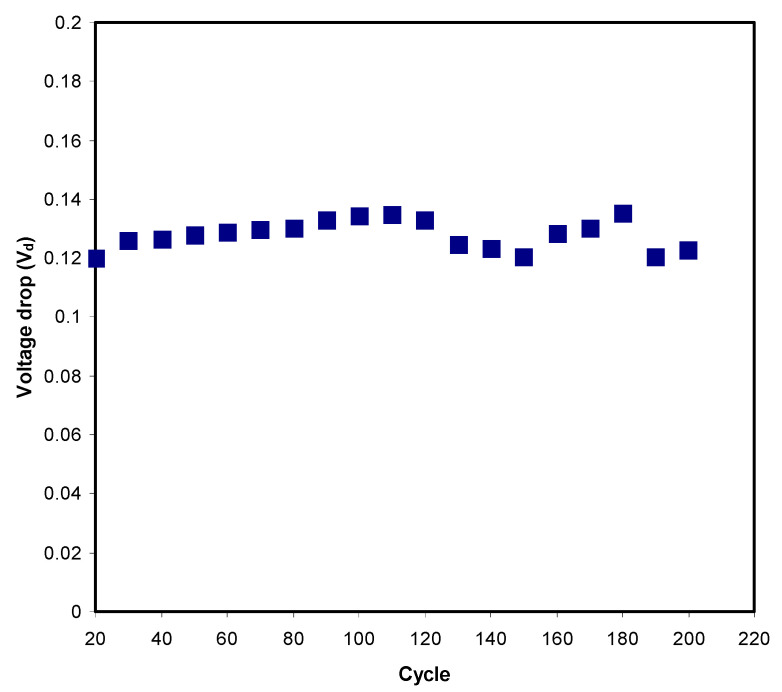
Voltage drop before discharge process for 200 cycles.

The typical charge–discharge plot of the EDLC is depicted in Figure 6. The constructed EDLC is subjected to a current density of 0.75 mA/cm^2^ from 0 to 0.9 V. The linearity of the slope indicates that the EDLC has the right capacitor energy storage mechanism [80]. Before the discharge process, it is noticeable that there is a slight sharp reduction in potential. This is due to several reasons, including electrolyte bulk resistance and the gap between the electrolyte and current collectors. The *V_d_* value for each cycle is plotted in Figure 7**,** where it is stable until the 120th cycle at an average of 0.125 V. Beyond the 120th cycle, a fluctuated pattern of *V_d_* can be seen. Equation (6) can be used to calculate equivalence series resistance (ESR) as V_d_ has been obtained. ESR values are tabulated in Table 6. The internal resistance of the EDLC is found to be <90 Ohm. The small value of ESR means that the EDLC in this work has good electrode–electrolyte contact where it is easy for ions to move towards the activated carbon electrode surface [81].

**Figure 8:**
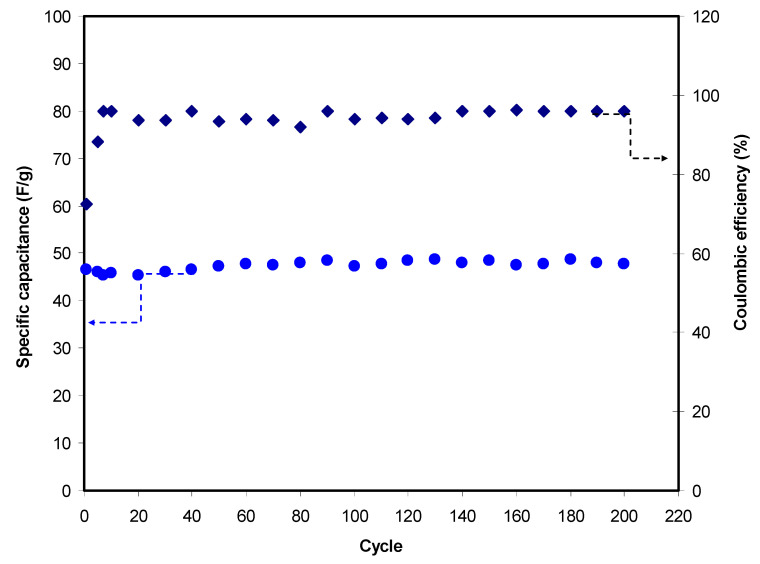
Specific capacitance and efficiency of the EDLC throughout the 200 cycles.

The value of *C_cd_* of the EDLC in this work is presented in Figure 8. A stable set of *C_cd_* is obtained with an average of 47.4 F/g throughout 200 cycles. The large surface area of activated carbon (2500 m^2^/g) allows charge carriers of Mg(CH_3_COO)_2_ to develop a charge double-layer. Chong et al. [82] reported that the inclusion of silica nano-particles into hydroxyethyl cellulose-Mg(CF_3_SO_3_)_2_ provides *C_cd_* of 25.1 F/g. The PVdF-HFP-MgTF2-EMITf system-based EDLC shows a range of *C_cd_* from 31 to 41 F/g [83]. The EDLC reported by Francis et al. [84] has a *C_cd_* value from 15 to 45 F/g for PVA-Mg(CF_3_SO_3_)_2_ system. The efficiency of the EDLC is illustrated in Figure 8. The efficiency of 72.5% can be seen in the 1st cycle. Typically, at initial cycles, the charging process takes a longer time compared to discharging. As current is applied, charge carriers start to migrate towards the electrode and become familiar with the conduction pattern. This stage usually gives low efficiency. The efficiency is 96% at the 7th cycle, where it is consistent until the 200th cycle at an average of 95%. A large efficiency signifies that the time taken for charging and discharge is almost identical. This outcome indicates that the EDLC in this work has good cycling stability and contact and less charge loss of up to 400 cycles.

**Figure 9:**
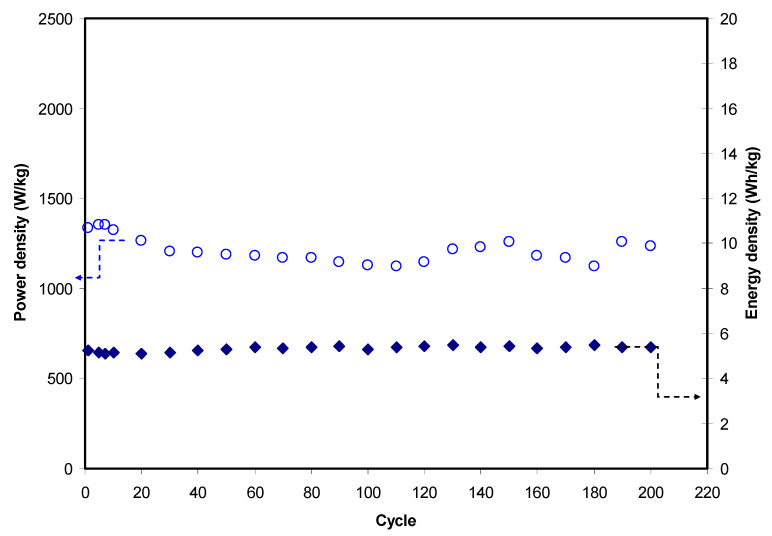
Energy and power density of the EDLC throughout the 200 cycles.

Figure 9 shows the variation of *E* and *P* for 200 cycles. E value is observed to be stable at an average of 5.32 Wh/kg throughout the 200 charge–discharge cycles. This outcome indicates that anions (CH_3_COO^−^) and cations (Mg^2+^) in the polymer chain of the CS–MC blend migrate toward the surface of the activated carbon electrode at an almost similar energy barrier [85]. By referring to Equation (7), energy density is directly proportional to specific capacitance; thus, *E* is expected to have a similar Ccd tren*_d_*. As reported by Bandaranayake et al. [86], a MgCl_2_-based EDLC provides E of 5 Wh/kg. Winie et al. [87] reported that a CS-based EDLC has energy density from 0.57 to 2.8 Wh/kg as the current density varies from 2 to 0.6 mA/cm^2^, respectively. A constant capacitance and energy density trend indicates that the number of ions aggregate formation is low. The EDLC has a higher power density than batteries due to the absence of the intercalation/deintercalation process in an EDLC. *P* of the EDLC in this work is also illustrated in Figure 9. *P*-value at the 1st cycle is 1338.5 W/kg and it drops to 1206.3 W/kg at the 30th cycle. According to work by Yassine et al. [88], power density is related to the internal resistance. The reduction of *P* is due to an increase in ESR value. Beyond the 40th cycle, the *P* pattern becomes more stable with a small fluctuation in the range of 1217 to 1261 W/kg. Bandaranayake et al. [86] reported a *P* of 341 W/kg.

## 4. Conclusions

CS:MC:Mg(CH_3_COO)_2_:Ni(II)-complex:glycerol based electrolytes were successfully synthesized via a solution-casting method. Chitosan was blended with methylcellulose for the host polymer, while ions were provided by magnesium acetate (Mg(CH_3_COO)_2_). Ni(II)-complex was added to improve the amorphous phase, while glycerol was used to improve ionic dissociation. The addition of 42 wt% glycerol maximized the conductivity to 1.02 × 10^−4^ S cm^−1^. The XRD results indicated that the maximum conducting plasticized electrolyte has the minimum crystallinity degree determined to be 2.08. The XRD outcomes can also be associated with the trend in the degree of crystallinity with the change in the electrolytes’ conductivity. It was identified that ions in CS:MC:Mg(CH_3_COO)_2_:Ni(II)-complex:glycerol were the dominant charge carrier as *t_i_* > *t_e_*_._ The electrolyte in this work can withstand applied potential up to 2.48 V; thus, the electrolyte was useful for EDLC applications. Capacitive characteristics in the assembled EDLC were verified by cyclic voltammetry, as no redox current was produced. Specific capacitance was found to reduce with increasing scan rate. Important parameters of the EDLC were stable up to 200 cycles. Energy density, specific capacitance and power density were 5.32 Wh/kg, 47.4 F/g and 1338.5 W/kg, respectively. Good cyclability of the EDLC was achieved as the value of efficiency was ~95%.

## Figures and Tables

**Table 1:** The designation and composition for the glycerol plasticized of chitosan (CS):methylcellulose (MC):Mg(CH_3_COO)_2_: Ni(II)-complex systems.

Sample Designation	CS (g)	MC (g)	Mg(CH_3_COO)_2_ (g)	Ni(II)-Complex (mL)	Glycerol (g)	Glycerol (wt%)
CSMCMNG1	0.5	0.5	0.666	10	0.271	14
CSMCMNG2	0.5	0.5	0.666	10	0.647	28
CSMCMNG3	0.5	0.5	0.666	10	1.206	42

**Table 2:** The degree of crystallinity using deconvoluted X-ray diffraction (XRD) examination.

Electrolyte	Degree of Crystallinity (%)
Pure CS	15.97
CS: MC	13.93
CSMCMNG1	11.21
CSMCMNG3	2.08

**Table 3:** The electrical equivalent circuit (EEC) fitting parameters for plasticized electrolyte systems at room temperature.

Sample	K_1_ (F^−1^)	K_2_ (F^−1^)	C_1_ (F)	C_2_ (F)
CSMCMNG1	9.6 × 10^8^	3.65 × 10^5^	1.04 × 10^−9^	2.74 × 10^−6^
CSMCMNG2	8.6 × 10^8^	2.65 × 10^5^	1.16 × 10^−9^	3.77 × 10^−6^
CSMCMNG3	-	1.0 × 10^5^	-	1.0 × 10^−5^

**Table 4:** DC conductivity for plasticized electrolyte systems at room temperature.

Designation	R_b_ (Ohm)	Conductivity (S cm^−1^)
CSMCMNG1	4.97 × 10^4^	2.31 × 10^−7^
CSMCMNG2	2.96 × 10^4^	4.42 × 10^−7^
CSMCMNG3	190	1.02 × 10^−4^

**Table 5:** Specific capacitance at various scan rates.

Scan Rate (mV/s)	*C_cv_* (F/g)
100	16.8
50	25.8
20	32.6
10	35.2

**Table 6:** Internal resistance of the EDLC at selected cycles.

Cycle Number	ESR (Ohm)
1	75.7
10	76.3
30	83.9
50	85.2
70	86.7
90	88.5
110	89.9
130	83.2
150	80.3
170	86.7
200	81.9
